# Colonic resection and stoma formation due to chronic diverticular disease: nationwide population-based cohort study

**DOI:** 10.1093/bjsopen/zraf008

**Published:** 2025-03-04

**Authors:** Helene Rask Dalby, Rune Erichsen, Kåre Andersson Gotschalck, Katrine Jøssing Emmertsen

**Affiliations:** Department of Surgery, Randers Regional Hospital, Randers, Denmark; Department of Clinical Epidemiology, Aarhus University Hospital, Aarhus, Denmark; Department of Surgery, Randers Regional Hospital, Randers, Denmark; Department of Clinical Epidemiology, Aarhus University Hospital, Aarhus, Denmark; Department of Surgery, Horsens Regional Hospital, Horsens, Denmark; Department of Clinical Medicine, Aarhus University, Aarhus, Denmark; Department of Surgery, Randers Regional Hospital, Randers, Denmark; Department of Clinical Medicine, Aarhus University, Aarhus, Denmark

## Abstract

**Background:**

Consensus on patient selection for elective colonic resection in patients with chronic diverticular disease is lacking. Early identification of patients who require surgery eventually enables timely elective resection, which could decrease the chronic diverticular disease burden. This register-based nationwide cohort study aimed to investigate the incidence of emergency and elective colonic resections or stoma formation in patients with chronic diverticular disease and explore predictors for surgery.

**Methods:**

The study included all patients with chronic diverticular disease in Denmark from 1996 to 2020, defined as patients with two or more hospital contacts due to diverticular disease. The incidence of surgery due to chronic diverticular disease was calculated as cumulative incidence proportions. Predictors for surgery were explored in a Cox proportional hazard model.

**Results:**

A total of 33 951 patients with chronic diverticular disease were included. The overall 5-year cumulative incidence proportion of surgery was 13.9% (elective surgery 9.8%, emergency surgery 4.2%). Patients with complicated chronic diverticular disease, including fistula, stenosis or perforation, had a three- to six-fold higher incidence of surgery overall than patients with uncomplicated chronic diverticular disease. The incidence of elective surgery decreased with age and co-morbidity and increased with the number of emergency admissions, even more pronounced if the emergency admissions accumulated within a shorter interval.

**Conclusion:**

Patients with chronic diverticular disease should be considered for elective colonic resection if they have complicated disease or several hospital contacts as they are likely to undergo surgery eventually.

## Teaser

In a register-based cohort study, we investigated colonic resection and/or stoma formation in patients with chronic diverticular disease. Of 33 951 patients, the 5-year cumulative incidence proportion of surgery overall was 13.9% (elective 9.8%, emergency 4.2%). Predictors for surgery overall included complicated disease and, for elective surgery specifically, low age, no co-morbidity and increasing number of emergency admissions.

## Introduction

In recent decades, admissions due to diverticular disease (DD) have increased by 26–65% in Western populations^1–4^. While the incidence is greatest among the elderly, the most marked increase in incidence is seen in the younger population^1,4–6^. If the initial episode of acute diverticulitis is treated without colonic resection, recurrence is 12–48%^1,7–12^. Recurrences, persisting symptoms or complications, including fistula or colonic stenosis, collectively referred to as chronic diverticular disease (cDD), evolve in some patients with DD. cDD includes a range of disabling symptoms, and several studies have demonstrated that elective resection may increase patients’ quality of life^8,9,13–15^. Indications for elective resection in cDD have changed towards an individualized single-patient evaluation. Guidelines argue that the only justifiable reason for elective resection is to improve quality of life^16–18^.

The individualized approach to elective resection creates a gap, leaving no consensus on whom to operate on electively. Early identification of patients who eventually require resection enables timely elective surgery, which could decrease the burden of cDD for patients and society and even the risk of emergency surgery. More studies are needed to support patient selection. A few older studies explore the long-term course after an initial episode of DD managed without colonic resection, none of which specifically evaluates the incidence of and predictors for surgery in cDD^10,19–23^. Contemporary population-based studies to validate current guidelines are lacking, and a deeper understanding of which patients with cDD undergo surgery is needed to support or revise these guidelines.

This study investigates the incidence of emergency and elective colonic resections or stoma formation in patients with cDD in Denmark from 1996 to 2020 within a population-based setting. In addition, potential predictors for surgery and changes in surgery for cDD during the past 25 years are investigated.

## Methods

### Setting and design

This nationwide registry-based cohort study includes all patients diagnosed with cDD in the Danish population (around 5.8 million inhabitants) between 1 January 1996 and 31 December 2020. The Danish national healthcare system provides universal tax-supported healthcare, guaranteeing patients’ access to general practitioners and hospitals. The unique personal civil registration (CPR) number assigned to all Danish citizens at birth or upon immigration allows for precise linkage of data sources at an individual level^24^. This study was based on anonymized registry data. It was therefore exempt from ethical committee review and informed consent per the General Data Protection Regulations. The study was registered at the Danish Data Protection Agency in the Central Denmark Region (record no. 2016–051-000001, 2624). The study was reported following the Strengthening the Reporting of Observational Studies in Epidemiology (STROBE) guideline^25^.

### Data sources

The Danish Civil Registration System (CRS) and the Danish National Registry of Patients (DNRP) were linked via the CPR number. The CRS contains individual-level data, for example sex, date of birth, emigration and date of death, with high validity and completeness^26,27^. The DNRP contains data on outpatient and inpatient hospital contacts and surgical procedures within all Danish hospitals since 1977. Contacts are registered as elective or emergency.

### Study cohort

Patients were identified through the DNRP based on their DD diagnoses. The positive predictive value of the DD diagnosis in the DNRP is 98%^27^. Patients entered the cohort when they fulfilled the cDD criteria (index date). Patients were defined as having cDD when they were registered with their second relevant hospital contact within 5 years from their first relevant hospital contact. Relevant hospital contacts were defined as outpatient or inpatient hospital (including emergency department) contacts registered with a primary diagnosis of either DD or complications to DD including colonic stenosis or fistula with a preceding diagnosis of DD. To avoid the inclusion of asymptomatic diverticulosis, patients were excluded that had outpatient contact with an endoscopic procedure. Patients with a colonic resection or stoma formation before their index date were excluded. Due to changes in the surgery coding classification in 1995, only patients with index dates from 1996 onwards were included. The end of follow-up was 31 December 2021, so to ensure a minimum 1-year risk time, patients with an index date until 31 December 2020 were included. The diagnosis and procedure codes used in the study are listed in *Table S1*.

### Outcome

The primary outcome was elective or emergency colonic resection or stoma formation due to cDD. The type of admission (that is elective or emergency) was applied to categorize the surgery. Surgical approach (minimally invasive surgery (MIS) or open) and rate of stoma formation were registered.

### Possible predictors

Information on possible predictors for surgery was extracted from the DNRP. The severity of cDD was classified as complicated if involving abscess, perforation, stenosis, or fistula at any point before or on the index date. Co-morbidity was classified according to the Charlson Co-morbidity Index (CCI)^28^ and estimated from all diagnoses coded before or on the index date. The time between the two consecutive contacts leading to cDD was investigated as a proxy for disease duration. The numbers of relevant hospital contacts and emergency admissions due to DD during follow-up (that is from index to outcome or end of follow-up) were explored.

### Follow-up

Patients were followed until the outcome defined as first colonic resection or stoma formation for cDD (surgery), surgery for reasons other than cDD, death, emigration or end of follow-up.

### Statistical analysis

#### Patients with cDD

Descriptive statistics were compiled using proportions for all patients. Quantitative data were estimated as median with interquartile (i.q.r.) range and categorical data as absolute numbers and percentages.

#### Incidence of surgery

Incidence rates (IR) of emergency and elective surgery were estimated as the proportion of surgeries relative to person-years at risk until the outcome and reported with 95% confidence intervals (c.i.). Cumulative incidence proportions (CIPs) at 1, 5 and 10 years were calculated as absolute measures for the incidence of surgery, treating surgery for reasons other than cDD and death as competing events, and emigration and end of follow-up as censoring events. CIP was stratified according to predictors, calculated according to admission type (emergency or elective) and reported with 95% c.i. CIP curves were constructed using the Aalen–Johansen estimator.

#### Predictors for surgery

The association between the predictors and emergency or elective surgery was calculated using the Cox proportional hazard model. Hazard ratios (HRs) were reported as cause-specific HRs with 95% c.i., mutually adjusted for the predictors at the index date. In the Cox analysis, surgery for reasons other than cDD, death, migration and end of follow-up were censoring events.

#### Significance of number of hospital contacts

The significance of contacts during follow-up was further explored in two subanalyses. Firstly, the CIP of emergency and elective surgery for patients with three or more (3+) or four or more (4+) relevant hospital contacts was calculated by changing the index date to the third or fourth contact. The CIP and HRs for surgery were estimated in the subanalyses as for the overall cohort.

In the second subanalysis, the association between the number of emergency admissions due to DD and the incidence of elective surgery was investigated by calculating the CIPs of elective surgery following each emergency admission. Patients were followed from each emergency admission at or after the index date and until elective surgery (outcome), emergency surgery or death (competing events), or new emergency admission for DD, emigration or end of follow-up (censoring events). In this model, patients with surgery at their index contact were excluded. If patients had a new emergency admission for DD, they were censored and then followed from that admission until the outcome, competing or new censoring event. This process was repeated for up to four emergency admissions. HRs were estimated as for the overall cohort.

#### Surgical procedures

Changes in procedures over the study interval were investigated with descriptive statistics.

Statistical analyses were conducted using RStudio (RStudio Team, Boston), v. 2022.12.0.353 (Posit PBC). Data were analysed from August 2023 to April 2024.

## Results

### Patients with cDD

**Fig. 1:**
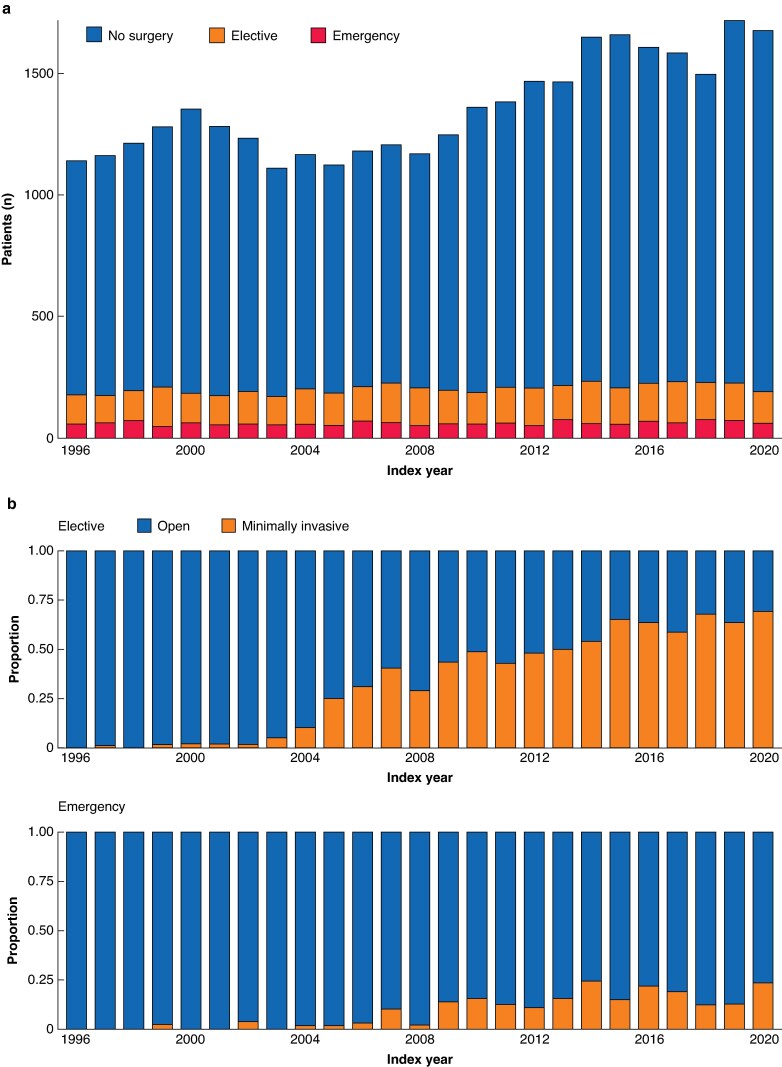
Surgical procedures over the study interval **a** Annual number of patients with chronic diverticular disease according to index year stratified by outcome (no surgery, elective surgery or emergency surgery). **b** Proportions of open *versus* minimally invasive surgical approach according to the year of the procedures, stratified by contact type (elective or emergency)

A total of 39 099 patients were identified with cDD from 1996 to 2020. After excluding 5148 patients who had a colonic resection or stoma formation before diagnosis of cDD, 33 951 (87%) patients were included in the study cohort. The annual number of patients diagnosed with cDD increased over the study interval from 1142 in 1996 to 1678 patients in 2020 (*Fig. 1a*). The median age at the index date of cDD was 68 (57–78) years, and 62% were female. Most patients had uncomplicated cDD (83%), and 85% had no or few co-morbidities (*Table 1*). The median follow-up time was 5 years^2–10^.

**Table 1:** Demographics of patients with chronic diverticular disease at index time

Demographics at index	Total (*n* = 33 951)
**Sex**	
Male	12 736 (38)
Female	21 215 (62)
**Age (years)**	
<50	4321 (13)
50–59	6090 (18)
60–69	7595 (22)
70–79	8695 (26)
80+	7250 (21)
**CCI score**	
0 (no co-morbidity)	16 726 (49)
1–2	11 998 (35)
3+	5227 (15)
**Index year**	
1996–2000	6155 (18)
2001–2005	5917 (17)
2006–2010	6168 (18)
2011–2015	7627 (22)
2016–2020	8084 (24)
**Time between contacts leading to cDD diagnosis**	
<30 days	12 654 (37)
30–364 days	14 353 (42)
1–5 years	6944 (20)
Severity of cDD*	
Uncomplicated	28 275 (83)
Complicated	5676 (17)
Subgroups of complicated cDD†	
Fistula	994 (3)
Stenosis	1226 (4)
Abscess/perforation	3757 (11)

Values are *n* (%). *Severity of cDD at index time, categorized as complicated if any contact before or at index contact were with abscess, perforation, stenosis or fistula. †One patient may have both fistula, stenosis and/or abscess/perforation. Consequently, one patient may be categorized within multiple subgroups of complicated cDD. CCI score, Charlson Co-morbidity Index score; cDD, chronic diverticular disease.

### Incidence of surgery

**Fig. 2:**
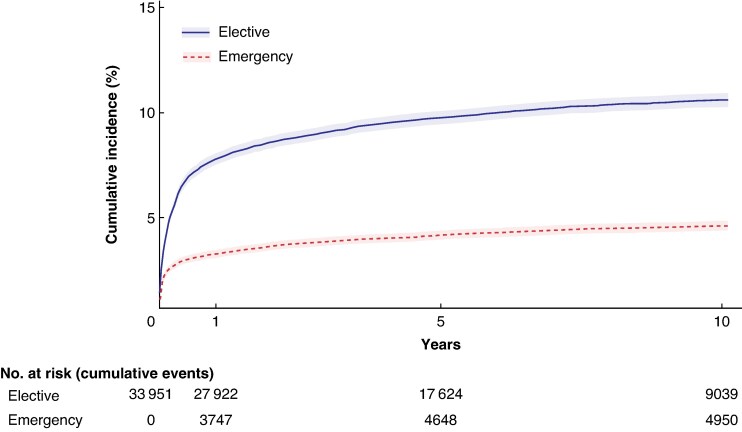
Cumulative incidence proportion of elective and emergency surgery for chronic diverticular disease

During the study interval, a total of 5088 (15%) patients with cDD had surgery performed, of which 70% (*n* = 3544) were elective procedures and 30% (*n* = 1544) were emergency procedures. The incidence rate of elective surgery was 4.1 (95% c.i. 4.0 to 4.2) per 100 000 person-years, and for emergency surgery was 1.8 (95% c.i. 1.7 to 1.9) per 100 000 person-years. The overall CIP was 7.8% at 1 year and 9.8% at 5 years for elective surgery; 3.3% and 4.2% for emergency surgery respectively (*Fig. 2* and *Table 2*). The CIPs at 5 and 10 years were similar and only 1- and 5-year CIPs are reported in *Table 2*.

**Table 2:** Cumulative incidence proportions (CIPs) (%) of surgery in patients with chronic diverticular disease (cDD) with colonic resection or stoma formation for reasons other than DD and death as competing events and death and migration as censoring events

		1 year		5 years
Eligible for follow-up	(*n* = 27 992)		(*n* = 17 624)	
	Emergency	Elective	Emergency	Elective
Overall	3.3 (3.1,3.5)	7.8 (7.5,8.1)	4.2 (4.0,4.4)	9.76 (9.4,10.1)
**Sex**				
Male	3.1 (2.8,3.4)	8.8 (8.3,9.3)	3.9 (3.6,4.2)	10.7 (10.1,11.2)
Female	3.4 (3.1,3.6)	7.2 (6.8,7.5)	4.3 (4.1,4.6)	9.2 (8.8,9.6)
**Age (years)**				
<50	2.6 (2.1,3.1)	10.8 (9.9,11.7)	3.6 (3.1,4.2)	14.7 (13.7,15.8)
50–59	3.0 (2.6,3.5)	10.4 (9.6,11.2)	4.0 (3.5,4.5)	13.7 (12.8,14.6)
60–69	3.6 (3.2,4.0)	10.1 (9.5,10.8)	4.6 (4.1,5.0)	12.4 (11.7,13.2)
70–79	3.8 (3.4,4.2)	7.2 (6.6,7.7)	4.8 (4.3,5.2)	8.4 (7.8,9.0)
80+	2.9 (2.6,3.4)	2.1 (1.8,2.5)	3.6 (3.1,4.0)	2.5 (2.1,2.9)
**CCI score**				
0	3.0 (2.7,3.2)	9.6 (9.1,10.0)	3.9 (3.6,4.2)	12.2 (11.7,12.7)
1–2	3.5 (3.2,3.9)	6.9 (6.4,7.4)	4.4 (4.1,4.8)	8.6 (8.1,9.1)
3+	3.6 (3.2,4.2)	4.1 (3.6,4.7)	4.4 (3.9,5.0)	4.9 (4.3,5.5)
**Index year**				
1996–2000	3.1 (2.7,3.6)	7.3 (6.7,8.0)	4.1 (3.6,4.6)	8.8 (8.1,9.5)
2001–2005	3.3 (2.9,3.8)	8.3 (7.6,9.0)	4.1 (3.6,4.6)	9.9 (9.1,10.7)
2006–2010	3.7 (3.2,4.1)	8.9 (8.2,9.6)	4.5 (4.0,5.0)	10.7 (9.9,11.5)
2011–2015	2.8 (2.4,3.2)	7.2 (6.7,7.8)	3.6 (3.2,4.1)	9.4 (8.8,10.1)
2016–2020	3.5 (3.1,3.9)	7.5 (6.9,8.0)	4.6 (4.1,5.1)	10.1 (9.4,10.9)
**Time between contacts leading to cDD diagnosis**				
<30 days	3.7 (3.4,4.1)	8.5 (8.1,9.0)	4.6 (4.2,5.0)	10.2 (9.6,10.7)
30–364 days	2.8 (2.6,3.1)	8.4 (8.0,8.9)	10.6 (10.1,11.1)	4.2 (3.9,4.6)
1–5 years	3.4 (3.0,3.8)	5.0 (4.5,5.5)	7.3 (6.7,7.9)	4.7 (4.2,5.3)
Severity of cDD*				
Uncomplicated	1.6 (1.5,1.8)	5.4 (5.2,5.7)	2.4 (2.2,2.6)	7.3 (7.0,7.6)
Complicated	11.4 (10.6,12.2)	19.5 (18.5,20.5)	12.9 (12.0,13.8)	22.1 (21.0,23.2)

Values are cumulative incidence proportion (%) with 95% confidence intervals. *Severity of cDD at index time, categorized as complicated if any contact before or at index contact were with abscess, perforation stenosis or fistula. CCI score, Charlson Co-morbidity Index score; cDD, chronic diverticular disease.

### Predictors for surgery

**Fig. 3:**
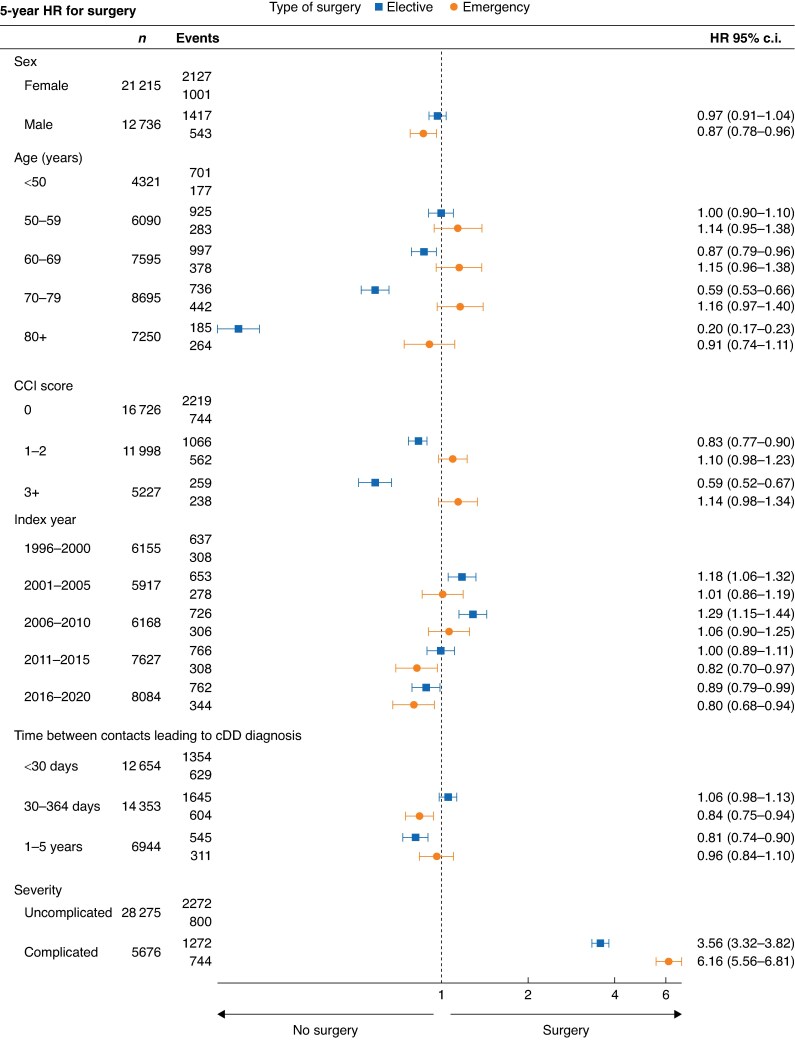
Forest plot showing adjusted cause-specific hazard ratios (HR) for emergency and elective surgery due to chronic diverticular disease (cDD)

The severity of cDD was the most significant predictor for surgery (*Fig. 3*). Patients with complicated cDD had a six-fold higher incidence of emergency surgery and more than a three-fold higher incidence of elective surgery than patients with uncomplicated cDD. Patients with index year after 2010 had a decreased incidence of surgery. The incidence of elective surgery remarkably declined with increasing age and CCI score. The incidence of elective surgery was lowest in patients with more than a year between the two contacts leading to cDD diagnosis, while the incidence of emergency surgery was lowest for patients with 30–364 days between the two contacts leading to cDD diagnosis. The HRs for surgery within 1, 5 and 10 years of diagnosis of cDD were similar. The adjusted 5-year HR is presented in *Fig. 3*, and unadjusted and adjusted 1-year and unadjusted 5-year HRs are given in *Table S2*.

CCI, Charlson co-morbidity index.

### Significance of number of hospital contacts

In the first subanalysis of the subgroups of patients with 3+ or 4+ relevant contacts, the 5-year CIPs of elective and emergency surgery were higher than in the overall study cohort; only slightly so for emergency surgery, but considerably so for elective surgery. For patients with 3+ contacts, the 5-year CIP of elective surgery was 15.1% (95% c.i. 14.6 to 15.7) and for emergency surgery was 5.2% (95% c.i. 4.9 to 5.6). For patients with 4+ contacts, the 5-year CIP was 20.3% (95% c.i. 19.5 to 21.1) for elective surgery and 5.8% (95% c.i. 5.4 to 6.3) for emergency surgery. The HRs for the predictors of elective and emergency surgery were consistent with the general findings.

**Fig. 4:**
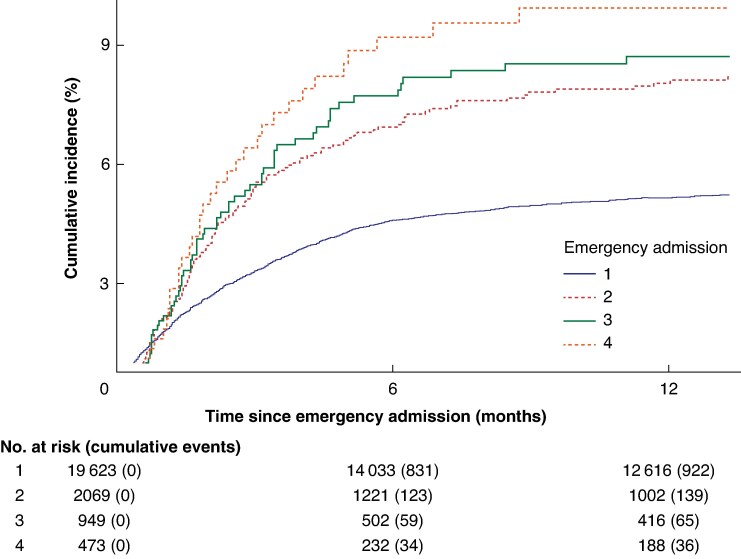
Cumulative incidence proportion of elective surgery only for chronic diverticular disease stratified by number of emergency admissions for diverticular disease at or after diagnosis of chronic diverticular disease

The second subanalysis of the CIP of elective surgery stratified by the number of emergency admissions revealed that it was notably higher after the second than after the first emergency admission after a diagnosis of cDD. The CIP of elective surgery only slightly increased at each subsequent emergency admission following the second admission after the index date (*Fig. 4*). The HR for elective surgery increased with each admission. The increase was even more pronounced after adjusting for time since diagnosis of cDD. Among patients with more than one emergency admission (*n* = 2069), after adjusting for the time since the last emergency admission, the likelihood of undergoing elective surgery decreased as the time from the recent emergency admission increased.

### Surgical procedures

Of the 3544 elective procedures, 39% were done by MIS and 18% included a stoma formation. Of the 1544 emergency procedures, 11% were done by MIS and 77% included a stoma formation. During the last decades of study, the proportion of MIS gradually increased, most so for elective procedures (*Fig. 1b*). Until 2004, the proportion of MIS was less than 5% of both emergency and elective procedures. From 2015 onwards, MIS comprised 19% of emergency and 60% of elective procedures. The proportion of stoma formation was stable over the study interval. Almost all colonic resections were left-sided (96%). The median age at the time of surgery was 62 years (53–71) for elective surgery and 69 (59–78) for emergency surgery. Among patients undergoing elective surgery, 65% had surgery more than 30 days after the index date, and 43% had at least two further hospital contacts before undergoing surgery. Among patients undergoing emergency surgery, 24% had surgery at the index contact, and 76% had no or only one further hospital contact after the index date before undergoing surgery.

## Discussion

This population-based study is the first to use a register-based definition of cDD and to investigate the incidence of and predictors for colonic resection and stoma formation in patients with cDD. The study reflects clinical practice and reports how patients with cDD have been managed in Denmark during the last 25 years. Within 5 years of a diagnosis of cDD, 9.8% underwent elective and 4.2% underwent emergency surgery.

The most notable predictor for surgery was complicated compared with uncomplicated cDD, underscoring the importance of carefully considering surgical treatment early after an initial episode of non-surgically managed complicated cDD. A high proportion of patients with complicated cDD will ultimately need surgery.

Older and co-morbid patients had the lowest incidence of elective surgery, whereas the incidence of emergency surgery remained relatively constant across all subgroups, regardless of age or co-morbidity. This aligns with a conservative approach to elective colonic resection, with frail patients typically undergoing surgery only in cases of acute, potentially life-threatening peritonitis. Frailty is associated with an increased risk of complications to both DD and after surgery. Additionally, morbidity and mortality rates are higher in the emergency setting^1,20,29^. Therefore, the potential of preventing emergency surgery through timely elective resection should be considered, making careful selection of patients for elective colonic resection crucial.

Patients with an index date after 2016 had a lower HR for elective surgery than those with an index date early in the study interval. This trend may be attributable to an increasingly restrictive approach towards elective surgery, reflecting a paradigm shift in clinical guidelines on the management of cDD that was implemented in Denmark in 2011^16^. Notably, this shift was not associated with a corresponding increase in emergency surgeries since the HR for emergency surgery was lowest among patients with an index date after 2010.

No precise definition of cDD exists, which makes the investigation of cDD in a register-based setting challenging. There were no data available from primary healthcare and therefore only patients who required secondary care in hospital were included. Nonetheless, cDD is known to affect patients without hospital contacts^30^. The total number of hospital contacts was assessed and associations between the predictors for surgery did not change, no matter how many contacts the patients had. The incidence of elective surgery increased with the number of hospital contacts overall and with emergency admissions only. Interestingly, the incidence increased significantly more when these admissions accumulated within a short interval rather than simply by their total number. Additionally, the incidence of elective surgery was higher in patients with less than a year between the two contacts leading to cDD diagnosis, compared with a longer time between the contacts. This pattern likely reflects a tendency to perform elective surgery in patients with several hospital contacts within a short time frame and is in line with previous studies reporting an increased risk of both emergency and elective surgery with subsequent admissions after an initial non-surgically managed emergency admission^10,19^. No studies have specifically reported if the time between contacts impacts the risk of surgery.

Only a few population-based studies have investigated the incidence of surgery in DD. Most studies only investigated the incidence of surgery at admissions with acute DD, either only reporting treatment at the first admission^31–33^ or reporting treatment at all admissions with acute DD, not considering if patients had their first or recurrent episode^2,3,34–36^. These studies report the rate of surgery at the initial admission to be 8–31%^2,3,31–33,35^ and up to 55% in patients with complicated DD^34^. Few studies secondarily report the incidence of surgery at recurrent admissions. One study found that 5.5% of 20 000 patients treated non-surgically at their first admission with DD required emergency surgery during follow-up and that the HR for emergency surgery doubled for each subsequent admission^19^. Two studies reported the rate of both elective and emergency surgery at recurrent admissions after an initial episode of conservatively managed acute DD^12,20^. The rate was 11–18% for emergency surgery and 3–9% for elective surgery. Studies investigating changes in the incidence of surgery over time report conflicting estimates: decreasing, stable and increasing numbers^2,3,32–35^. Previous studies have reported stoma rates of 5–21% in elective surgery^9,14,37^ and 56% in emergency procedures^2,19^, which is in line with the findings in the present study.

The strengths of the present study include a nationwide approach, reflecting general management of cDD for all patients, including older and co-morbid patients often not found eligible for inclusion in RCTs. The study reflects the actual management of cDD in Denmark during the last 25 years. The sample size was large, and all data were available for the entire cohort as all Danish citizens have unrestricted access to public tax-supported healthcare^38^. Management of cDD in Denmark adheres to European Guidelines^18^, suggesting that the findings of this study are generalizable to other countries with similar healthcare systems.

In this study of emergency and elective colonic resections or stoma formation in cDD, predictors for surgery were complicated cDD and several hospital contacts. Older and co-morbid patients less frequently undergo elective colonic resection or stoma formation. This is in line with an expected reluctance to perform elective surgery on frailer patients. The incidence of emergency surgery was consistent across age groups and co-morbidity levels, suggesting that older and co-morbid patients with cDD are equally at risk of life-threatening complications from DD. These findings may inform individualized single-patient management of cDD, assist in patient counselling and guide the selection of patients for elective colonic resection.

The limitations of this study include those that accompany the use of registry data. First, coding errors could lead to misclassification. However, the Danish registers have been validated previously and shown to have high reliability and completeness of data. Second, the definition of cDD as two contacts may cause an underestimation of the incidence of surgery in cDD if some patients in the cohort are not truly chronically affected, or an overestimation of the incidence of surgery if chronically affected patients are handling their symptoms without hospital contact. Third, the lack of ability to access more detailed clinical data such as smoking status or body mass index may lead to unmeasured confounding.

## Supplementary Material

zraf008_Supplementary_Data

## Data Availability

Study data cannot be made available due to the Danish General Data Protection Regulations. Researchers from certified Danish research institutions may access the databases used in this study by contacting Statistics Denmark (dst@dst.dk).

## Supplementary material

Supplementary  material is available at *BJS Open* online.
