# Supplementary material for: Colonic resection and stoma formation due to chronic diverticular disease: nationwide population-based cohort study
Source: BJS Open. 2025 Mar 4;9(2):zraf008. doi: 10.1093/bjsopen/zraf008 (PMC11879527; doi:10.1093/bjsopen/zraf008)
Supplement: zraf008_Supplementary_Data [file zraf008_supplementary_data.docx]

Colonic resection and stoma formation due to chronic diverticular disease: Nationwide population-based cohort study

Helene Rask Dalby, MD* ^1, 2^, Rune Erichsen, PhD^1, 2^, Kåre Andersson Gotschalck, PhD^3, 4^, Katrine J. Emmertsen, PhD^1,4^

1) Department of Surgery, Randers Regional Hospital, Randers, 2) Department of Clinical Epidemiology, Aarhus University Hospital, Aarhus, 3) Department of Surgery, Horsens Regional Hospital, Horsens, 4) Department of Clinical Medicine, Aarhus University, Aarhus, Denmark

**Corresponding author:**

Helene Rask Dalby, Randers Regional Hospital, Department of Surgery, Skovlyvej 15, 8930 Randers, Denmark

**ORCID ID**; 0000-0001-8923-314X

**X: @**HeleneRDalby

**Supplementary Materials - Index**

| **Supplementary Figures and Tables** |  |
| --- | --- |
| Table S1 | *pag. 2* |
| Table S2 | *pag. 3* |
| Table S3 | *pag. 4* |
|  |  |

## **Table S1:** Diagnosis and procedure codes used in the study

| **Diagnosis** | | ICD-8 (before 1994) | ICD-10 (from 1994 onwards) |
| --- | --- | --- | --- |
| **Diverticular disease and procedures** | | | |
| Diverticulosis | | 5621x | K572-9 |
| Diverticular disease | Uncomplicated | 56210, 56211, 56218, 56219 | K573, K575, K579 |
|  | Complicated | 56212, stenosis, fistula | K272, K574, K578, stenosis, fistula |
| Colonic stenosis | | 56091 | K566, K566A + G, K624B + E |
| Fistula (colovesical, colovaginal, colointestinal) | | 56910, 59601, 59905, 59906, 62989 | K632B, K632I, K632J, N321, N321A, N322, N823, N823A, N824 |
| **Comorbidities for Charlson Comorbidity Index score** | | | |
| Myocardial infarction | | 410 | I21, I22, I23 |
| Congestive heart failure | | 42709-11, 42719, 42899, 78249 | I50, I110, I130, I132 |
| Peripheral vascular disease | | 440, 441, 442, 443, 444, 445 | I70, I71, I72, I73, I74, I77 |
| Cerebrovascular disease | | 430-438 | I60-69, G45, G46 |
| Dementia | | 29009-20919, 29309 | F00-03, F051, G30 |
| Chronic pulmonary disease | | 490-493, 515-518 | J40-47, J60-67, J684, J701, J703, J841, J920, J961, J982, J983 |
| Connective tissue disease | | 712, 716, 734, 446, 13599 | M05-06, M08, M09, M30, M31, M32, M33, M34, M35, M36, D86 |
| Ulcer disease | | 53091, 53098, 531-534 | K221, K25-K28 |
| Mild liver disease | | 571, 57301, 57304 | B18, K700-03, K709, K71, K73-74, K760 |
| Diabetes type 1 and type 2 | | 24900, -06, -07, -09, 25000, -06, -07, -09 | E100-101, E109, E110-111, E119 |
| Diabetes with end organ damage | | 24901-05, 24908, 25001-05, -08 | E102-108, E112-118 |
| Hemiplegia | | 344 | G81, G82 |
| Moderate to severe renal disease | | 403, 404, 580-84, 59009, 59319, 75310-19, 792 | I12-13, N00-N05, N07, N11, N14, N17-N19, Q61 |
| Any tumor | | 140-194 | C00-C75 |
| Leukemia | | 204-207 | C91-C95 |
| Lymphoma | | 200-203, 27559 | C81-C85, C88, C90, C96 |
| Moderate to severe liver disease | | 07000, -02, -04, -06, -08, 57300, 45600-45609 | B150, B160, B162, B190, K704, K72 K766, I85 |
| Metastatic solid tumor | | 195-198, 199 | C76-C80 |
| AIDS | | 07983 | B21-B24 |
| **Procedure** | | **Surgeries before 1996** | **NOMESCO (from 1996 onwards)** |
| Colonic resection | | 44920-21, -60-61, 44980-81, 45020-21, -60-61, -80, -81, 45700, -20, -40, 45840-41, 46400, -40, -50, 46530, 46490 | JFB2-9, JGB, JFH |
| Stoma formation | | 45180, 45200, -01, -10, -40, 47000, -100, -110 | JFF |
| Endoscopies ^a^ | | 91000, -10, -20, 91070, -80, 92260, -80, 92300, -40, -60, 92490, 93160, -170, -210 | UJD, UJF, JFA, JCA, JDA, JDB10 |
| Abdominal wall, herniotomies, and mammary glands ^a^ | | *NA* ^b^ | JAA, JAC, JAD, JAF, HAx, HWx |

**a)** Used to exclude outpatient contacts considered as irrelevant to diagnose chronic diverticular disease

**b)** Considered irrelevant before 1996 since registration of out-patient surgery was after 1996

## **Table S2:** Crude and adjusted hazard ratios (HR) for risk of surgery in chronic diverticular disease (cDD).

|  | **1 year** | | | | **5 years** | |
| --- | --- | --- | --- | --- | --- | --- |
| *Contact type* | **Emergency** | | **Elective** | | **Emergency** | **Elective** |
| *Estimate* | *Crude* | Adjusted | *Crude* | Adjusted | *Crude* | *Crude* |
| **Sex** |  |  |  |  |  |  |
| Female | — | — | — | — | — | — |
| Male | 0.93 (0.84-1.04) | 0.85 (0.76-0.94) | 1.15 (1.07-1.23) | 0.94 (0.88-1.01) | 0.93 (0.84-1.03) | 1.14 (1.07-1.22) |
| **Age** (years) |  |  |  |  |  |  |
| < 49 | — | — | — | — | — | — |
| 50-59 | 1.07 (0.88-1.29) | 1.09 (0.91-1.32) | 0.88 (0.80-0.97) | 0.95 (0.86-1.05) | 1.11 (0.92-1.34) | 0.92 (0.84-1.02) |
| 60-69 | 1.02 (0.85-1.22) | 1.03 (0.85-1.23) | 0.68 (0.61-0.74) | 0.77 (0.70-0.85) | 1.15 (0.96-1.37) | 0.77 (0.70-0.85) |
| 70-79 | 0.90 (0.75-1.07) | 0.95 (0.79-1.14) | 0.37 (0.33-0.41) | 0.47 (0.42-0.52) | 1.15 (0.96-1.37) | 0.49 (0.44-0.54) |
| 80+ | 0.65 (0.53-0.79) | 0.69 (0.56-0.85) | 0.11 (0.09-0.13) | 0.14 (0.12-0.17) | 0.89 (0.74-1.08) | 0.16 (0.13-0.19) |
| **CCI-score** |  |  |  |  |  |  |
| 0 | — | — | — | — | — | — |
| 1-2 | 1.01 (0.91-1.13) | 1.06 (0.94-1.18) | 0.64 (0.60-0.69) | 0.79 (0.74-0.86) | 1.09 (0.97-1.21) | 0.69 (0.64-0.74) |
| 3+ | 1.02 (0.88-1.18) | 1.07 (0.92-1.25) | 0.37 (0.32-0.42) | 0.53 (0.47-0.61) | 1.14 (0.99-1.32) | 0.41 (0.36-0.47) |
| **Index year** |  |  |  |  |  |  |
| 1996-2000 | — | — | — | — | — | — |
| 2001-2005 | 1.10 (0.94-1.30) | 1.06 (0.90-1.25) | 1.21 (1.09-1.36) | 1.26 (1.13-1.41) | 1.06 (0.90-1.25) | 1.16 (1.04-1.30) |
| 2006-2010 | 1.37 (1.16-1.61) | 1.21 (1.03-1.43) | 1.49 (1.34-1.67) | 1.52 (1.36-1.69) | 1.22 (1.04-1.43) | 1.34 (1.20-1.49) |
| 2011-2015 | 1.31 (1.11-1.55) | 1.05 (0.89-1.24) | 1.47 (1.32-1.64) | 1.32 (1.18-1.48) | 1.03 (0.88-1.22) | 1.17 (1.05-1.31) |
| 2016-2020 | 1.62 (1.37-1.91) | 1.17 (0.98-1.38) | 1.59 (1.42-1.78) | 1.30 (1.16-1.46) | 1.11 (0.94-1.30) | 1.12 (1.00-1.24) |
| **Time between contacts leading to cDD diagnosis** |  |  |  |  |  |  |
| <30 days | — | — | — | — | — | — |
| 30-364 days | 0.85 (0.76-0.95) | 0.84 (0.75-0.94) | 1.07 (1.00-1.15) | 1.05 (0.98-1.13) | 0.84 (0.76-0.94) | 1.06 (0.99-1.14) |
| 1-5 years | 0.94 (0.82-1.08) | 0.96 (0.84-1.11) | 0.76 (0.69-0.84) | 0.81 (0.73-0.89) | 0.92 (0.81-1.06) | 0.74 (0.67-0.82) |
| **Severity** ^a^ |  |  |  |  |  |  |
| Uncomplicated | — | — | — | — | — | — |
| Complicated | 6.15 (5.57-6.80) | 6.03 (5.45-6.68) | 3.75 (3.50-4.02) | 3.46 (3.22-3.71) | 5.98 (5.41-6.61) | 3.65 (3.41-3.91) |

Numbers are hazard ratio (HR) with 95% confidence intervals

CCI score: Charlson Comorbidity Index score; cDD: Chronic diverticular disease

1. Severity of cDD at index time, categorised as complicated if any contact before or at index contact were with abscess, perforation, stenosis, or fistula

## Table S3: STROBE Checklist of items that should be included in reports of ***cohort studies***

|  | Item No | Recommendation | Page |
| --- | --- | --- | --- |
| **Title and abstract** | 1 | (*a*) Indicate the study’s design with a commonly used term in the title or the abstract | Title page |
|  |  | (*b*) Provide in the abstract an informative and balanced summary of what was done and what was found | 1 |
| Introduction | | |  |
| Background/rationale | 2 | Explain the scientific background and rationale for the investigation being reported | 2 |
| Objectives | 3 | State specific objectives, including any prespecified hypotheses |  |
| Methods | | |  |
| Study design | 4 | Present key elements of study design early in the paper | 4 |
| Setting | 5 | Describe the setting, locations, and relevant dates, including periods of recruitment, exposure, follow-up, and data collection | 4 |
| Participants | 6 | (*a*) Give the eligibility criteria, and the sources and methods of selection of participants. Describe methods of follow-up | 4 |
|  |  | (*b*) For matched studies, give matching criteria and number of exposed and unexposed | NA |
| Variables | 7 | Clearly define all outcomes, exposures, predictors, potential confounders, and effect modifiers. Give diagnostic criteria, if applicable | 5 |
| Data sources/ measurement | 8 | For each variable of interest, give sources of data and details of methods of assessment (measurement). Describe comparability of assessment methods if there is more than one group | 4 |
| Bias | 9 | Describe any efforts to address potential sources of bias | 5 |
| Study size | 10 | Explain how the study size was arrived at | 4-5 |
| Quantitative variables | 11 | Explain how quantitative variables were handled in the analyses. If applicable, describe which groupings were chosen and why | 5-6 |
| Statistical methods | 12 | (*a*) Describe all statistical methods, including those used to control for confounding | 6-7 |
|  |  | (*b*) Describe any methods used to examine subgroups and interactions |  |
|  |  | (*c*) Explain how missing data were addressed |  |
|  |  | (*d*) If applicable, explain how loss to follow-up was addressed |  |
|  |  | (*e*) Describe any sensitivity analyses |  |
| Results | | |  |
| Participants | 13 | (a) Report numbers of individuals at each stage of study—eg numbers potentially eligible, examined for eligibility, confirmed eligible, included in the study, completing follow-up, and analysed | 8 |
|  |  | (b) Give reasons for non-participation at each stage |  |
|  |  | (c) Consider use of a flow diagram |  |
| Descriptive data | 14 | (a) Give characteristics of study participants (eg demographic, clinical, social) and information on exposures and potential confounders | 8  Table 1 |
|  |  | (b) Indicate number of participants with missing data for each variable of interest | NA |
|  |  | (c) Summarise follow-up time (eg, average and total amount) | 8 |
| Outcome data | 15 | Report numbers of outcome events or summary measures over time | 8 |
| Main results | 16 | (*a*) Give unadjusted estimates and, if applicable, confounder-adjusted estimates and their precision (eg, 95% confidence interval). Make clear which confounders were adjusted for and why they were included | 8-9  Figure 1-3  Table 2  Table S2 |
|  |  | (*b*) Report category boundaries when continuous variables were categorized |  |
|  |  | (*c*) If relevant, consider translating estimates of relative risk into absolute risk for a meaningful time period |  |
| Other analyses | 17 | Report other analyses done—eg analyses of subgroups and interactions, and sensitivity analyses | 9  Figure 4 |
| Discussion | | |  |
| Key results | 18 | Summarise key results with reference to study objectives | 11 |
| Limitations | 19 | Discuss limitations of the study, taking into account sources of potential bias or imprecision. Discuss both direction and magnitude of any potential bias | 14 |
| Interpretation | 20 | Give a cautious overall interpretation of results considering objectives, limitations, multiplicity of analyses, results from similar studies, and other relevant evidence | 11-14 |
| Generalisability | 21 | Discuss the generalisability (external validity) of the study results | 13 |
| Other information | | |  |
| Funding | 22 | Give the source of funding and the role of the funders for the present study and, if applicable, for the original study on which the present article is based | Title page |
